# ADP ribosylation factor-like GTPase 6-interacting protein 5 (ARL6IP5): a prenylated Rab acceptor protein 1 (PRA1) family protein that shapes the ER membrane and regulates ER-phagy

**DOI:** 10.1080/27694127.2025.2513466

**Published:** 2025-06-10

**Authors:** Yasunori Yamamoto, Toshiaki Sakisaka

**Affiliations:** Division of Membrane Dynamics, Department of Physiology and Cell Biology, Kobe University School of Medicine, Kobe, Japan

**Keywords:** Endoplasmic reticulum, ER-phagy, prenylated Rab acceptor protein 1 domain, reticulon-homology domain, ARL6IP5, ARL6IP1

## Abstract

The prenylated Rab acceptor protein 1 (PRA1) domain is a conserved domain encompassing four transmembrane domains (TMDs). ARL6IP5 (ADP ribosylation factor-like GTPase 6-interacting protein 5) is a member of the PRA1 family and interacts with the reticulon-homology domain (RHD)-containing proteins including ARL6IP1 (ADP ribosylation factor-like GTPase 6-interacting protein 1) and FAM134B. The RHD is a conserved domain encompassing two short hairpin TMDs and acts as a membrane-shaping unit for endoplasmic reticulum (ER) morphology and ER-phagy. However, the involvement of ARL6IP5 in ER morphology and ER-phagy remains unclear. We recently characterized ARL6IP5 as an ER membrane-shaping protein and found that ARL6IP5 constricts the ER membrane in a manner similar to ARL6IP1, possibly via short hairpin configuration of the TMDs in the PRA1 domain. ARL6IP5 also plays a redundant role with ARL6IP1 in shaping the ER membrane. Importantly, depletion of ARL6IP5 impaired FAM134B-meadited ER-phagy, which is reminiscent of ARL6IP1 depletion. These results suggested that ARL6IP5 acts as an ER membrane-shaping protein that regulates ER-phagy, underscoring the importance of the PRA1 domain. Although ARL6IP5 and ARL6IP1 are confusable protein names and seem to have similar roles in ER-phagy, we clarify in this punctum that they are distinct classes of ER membrane-shaping proteins.

ER-phagy is the selective autophagy of the endoplasmic reticulum (ER) and it is important to maintain ER homeostasis. ER-phagy employs several ER membrane-shaping proteins containing the reticulon-homology domain (RHD) to fragment and target the ER by autophagy. For example, FAM134B (family with sequence similarity 134, member B), an RHD-containing protein, acts as an ER-phagy receptor by binding to LC3 on the phagophore membrane via the LIR (LC3-interacting region) motif. ARL6IP1 (ADP ribosylation factor-like GTPase 6-interacting protein 1) is another RHD-containing protein, which we originally identified as an ER membrane-shaping protein, and forms a heteromeric complex with FAM134B during autophagosome formation, leading to enhancement of ER-phagy. The RHD is a conserved structural domain characterized by two short hairpin transmembrane domains (TMDs). The RHD inserts the short hairpin TMDs into the outer leaflet of the lipid bilayer and forms oligomers, which in turn expand the area of the outer leaflet relative to the inner leaflet, leading to membrane constriction. RHD-containing proteins such as reticulons, REEPs (receptor expression-enhancing proteins) and ARL6IP1 exert the membrane-constricting activity generating ER tubules and the edges of ER sheets, both of which have high membrane curvature. In ER-phagy, the membrane-constricting activities of FAM134B and ARL6IP1 are utilized to drive ER membrane scission necessary for the sequestration of ER within autophagosomes.

The prenylated Rab acceptor protein 1 (PRA1) domain is a conserved domain homologous to PRA1 and encompasses four TMDs. Mammals have three PRA1 domain-containing proteins, PRA1, PRAF2 (PRA1 domain family, member 2), and ARL6IP5 (ADP ribosylation factor-like GTPase 6-interacting protein 5), which comprise the PRA1 family (Figure 1). PRA1 localizes at the Golgi membrane, whereas PRAF2 and ARL6IP5 localize at the ER membrane. PRA1 has been characterized as a GDI (Rab GDP dissociation inhibitor)-displacement factor that mediates the cycling of Rab GTPases between membrane-bound and soluble forms. However, it remains unknown whether PRAF2 and ARL6IP5 act as GDI-displacement factors. Interestingly, previous studies have shown that ARL6IP5 binds to ARL6IP1 and FAM134B. In addition, although the PRA1 domain does not have sequence similarity to RHD, it has been deduced that the TMDs in the PRA1 domain would adopt a short hairpin configuration. Although these results raise the possibility that ARL6IP5 would play roles in shaping the ER membrane and ER-phagy, no studies have examined this scenario. In our recent investigation [1], we characterized ARL6IP5 as an ER membrane-shaping protein. Upon overexpression, ARL6IP5 induced the formation of tubular ER and strongly constricted the ER membrane, thereby allowing the cells to maintain the ER tubules in the absence of microtubules. These phenotypes are reminiscent of overexpression of RHD-containing proteins. Upon disruption of the possible short hairpin structures of the PRA1 domain, ARL6IP5 is unable to constrict membranes. Consistently with these observations, ARL6IP5 depletion impaired the formation of reticular ER, which was rescued by ectopic expression of ARL6IP1. Furthermore, ARL6IP5 depletion inhibited impaired the ER-phagy flux mediated by FAM134B, which was reminiscent with ARL6IP1 depletion. These results indicated that ARL6IP5 acts as an ER membrane-shaping protein that regulates ER-phagy, underscoring the importance of the PRA1 domain in these processes.

**Figure 1.**
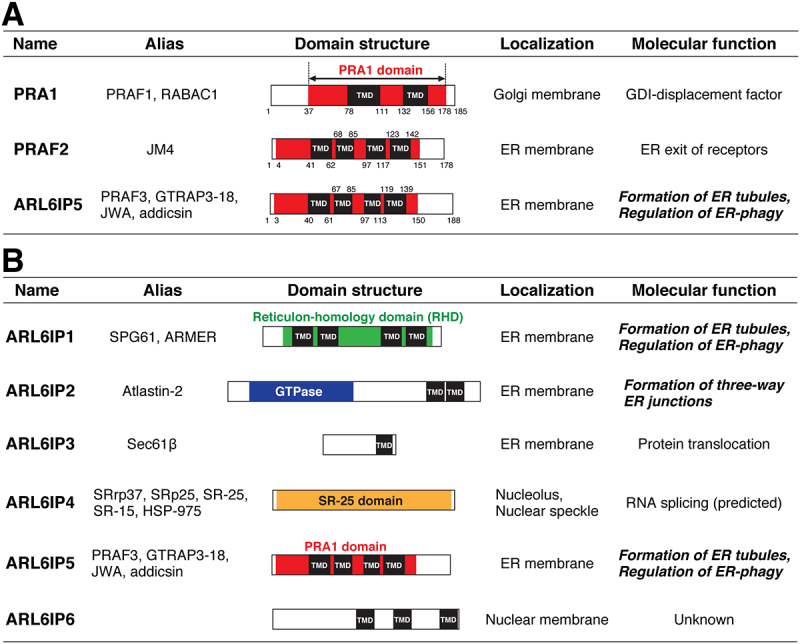
PRA1 family proteins and ADP ribosylation factor-like GTPase 6-interacting proteins (ARL6IPs). (**A**) Human PRA1 family proteins. The positions of the PRA1 domain and TMDs are based on SMART (https://smart.Embl.de) and phobius (https://phobius.Sbc.su.se/), respectively. Note that PRA1 is predicted to have two TMDs, presumably because phobius recognizes two tandem TMDs as a single, long TMD. (**B**) ARL6IPs. ARL6IPs are not a protein family but they are a collection of various proteins. In ARL6IP1, two tandem TMDs constitute a single short hairpin TMD. Of note, ARL6IP1, ARL6IP2/Atlastin-2 and ARL6IP5 are associated with ER remodeling.

As one can surmise from the names, Arl6IP5 and Arl6IP1 have been identified as proteins that bind to ARL6 (ADP ribosylation factor-like GTPase 6). To date, six ARL6IPs have been identified (Figure 1). Notably, ARL6IP2 is Atlastin-2, an ER-localized GTPase that fuses ER tubules into three-way junctions. Given that ARL6 is a regulator of protein transport in primary cilia, it is somewhat surprising that three of the six ARL6IPs are associated with ER remodeling. Although the possible binding of ARL6 to these proteins has been ignored so far, we cannot rule out the possibility that ARL6 might play an unknown role in ER remodeling. As mentioned above, ARL6IP1 is the ER membrane-shaping protein that we characterized more than a decade ago, and regulates FAM134B-mediated ER-phagy. We recently characterized ARL6IP5 as an ER membrane-shaping protein that regulates FAM134B-mediated ER-phagy [1]. Although both proteins appear to have similar properties, ARL6IP5 and ARL6IP1 rely on the PRA1 domain and the RHD, respectively, for their ER remodeling function. In this context and despite their name, ARL6IP5 and ARL6IP1 are distinct ER membrane-shaping proteins. Nevertheless, earlier studies often wrongly referred to ARL6IP5 as an RHD-containing protein because of the similarity in names. We would like to clarify here that ARL6IP does not refer to a conserved protein family but a collection of ARL6-interacting proteins (Figure 1), and emphasize that ARL6IP5 is a PRA1 domain-containing protein and not an RHD-containing protein.

Although the mechanism by which ARL6IP5 regulates ER-phagy remains unclear, our findings raise the possibility that ARL6IP5 could facilitate FAM134B-mediated membrane scission in a manner similar to ARL6IP1. Our findings also seem to imply that similarly to RHD, the PRA1 domain might act as a general membrane-shaping unit. If this is indeed the case, one could expect that PRA1 and PRAF2 also remodel Golgi and ER membranes, respectively. In particular, PRA1 might be involved in membrane remodeling for Golgi-phagy, although it is unknown whether PRA1 binds to Golgi-phagy receptors such as YIPF3 (Yip1 domain family, member 3) and YIPF4 (Yip1 domain family, member 4). Further studies are required to address these hypotheses.

## Data Availability

Data sharing is not applicable to this article as no data were created or analyzed in this study.

## Abbreviations

ARL6: ADP ribosylation factor-like GTPase 6
ARL6IP1: ADP ribosylation factor-like GTPase 6-interacting protein 1
ARL6IP5: ADP ribosylation factor-like GTPase 6-interacting protein 5
ER: endoplasmic reticulum
FAM134B: family with sequence similarity 134, member B
GDI: Rab GDP dissociation inhibitor
LIR: LC3-interacting region
PRA1: prenylated Rab acceptor protein 1
PRAF2: PRA1 domain family, member 2
REEP: receptor expression-enhancing protein
RHD: reticulon-homology domain
TMD: transmembrane domain
YIPF3: Yip1 domain family, member 3
YIPF4: Yip1 domain family, member 4
